# Effects of Tobacco Consumption and Anxiety or Depression during Pregnancy on Maternal and Neonatal Health

**DOI:** 10.3390/ijerph17218138

**Published:** 2020-11-04

**Authors:** Beatriz Pereira, Bárbara Figueiredo, Tiago Miguel Pinto, M. Carmen Míguez

**Affiliations:** 1 Department of Clinical Psychology and Psychobiology, Faculty of Psychology, Campus Vida, University of Santiago de Compostela, 15782 Santiago de Compostela, Spain; beatriz.pereira@usc.es; 2 Campus de Gualtar, School of Psychology, University of Minho, 4710-057 Braga, Portugal; bbfi@psi.uminho.pt (B.F.); tmpinto@psi.uminho.pt (T.M.P.)

**Keywords:** tobacco consumption, anxiety, depression, pregnancy complications, delivery complications, neonate’s health problems, low birth weight

## Abstract

This study analyzed the possible interaction effects between tobacco consumption and anxiety or depression during pregnancy on maternal and neonatal health. We recruited a sample of 807 pregnant Spanish women from public healthcare services. Women completed a questionnaire on sociodemographic variables, health status and tobacco consumption (continuous, quitting or no consumption) in the first and third trimester of pregnancy and at 2 months postpartum, and self-reported measures of anxiety and depression in the first trimester. Abstinence of tobacco consumption was verified through biochemical measurements. Interaction effects between tobacco consumption and anxiety were found for delivery (*p* < 0.001), neonatal health complications (*p* = 0.026) and gestational age at birth (*p* = 0.029). Interaction effects between tobacco consumption and depression were found for pregnancy (*p* = 0.032), delivery complications (*p* < 0.001) and weeks of gestation at birth (*p* = 0.031). This study suggests that there are different kinds of interaction effects between tobacco consumption and anxiety or depression. Smokers with high anxiety presented more delivery complications compared to quitters and non-smokers with high anxiety. There is a cumulative effect of anxiety on the effects of tobacco consumption on maternal health. The results highlighted the beneficial impact of quitting smoking during pregnancy to reduce the risk of suffering anxiety, depression and health complications.

## 1. Introduction

Maternal tobacco consumption during pregnancy has been linked with several maternal and infant health complications. Particularly, maternal tobacco consumption during pregnancy was associated with an increased risk of placental pathologies, such as ruptures or premature detachment of the membranes [1], placenta previa [2,3,4], ectopic pregnancy [5,6,7] and miscarriage [8,9]. Tobacco consumption during pregnancy is also a risk factor for neonatal health. Premature birth [10,11] and low birth weight are usually present, as the neonates of mothers who smoked during pregnancy weigh approximately 150–250 g less than those of non-smoking mothers [12]. Additionally, the risk of intrauterine growth restriction, fetal death, perinatal mortality (e.g., sudden death syndrome and respiratory distress syndrome), malformations and developmental delays is also increased in the neonates of mothers who smoked during pregnancy [11,13,14].

Anxiety and/or depression during pregnancy were associated with maternal and infant health complications that were similar to those produced by tobacco consumption, including preeclampsia, fetal distress, forceps delivery and prolonged or preterm labor [15,16,17,18]. High levels of maternal anxiety or depression during pregnancy were also associated with neonatal health [19,20,21], specifically preterm birth and low birth weight [21,22,23,24,25]. As stated by [19], anxiety and depression influence obstetric outcomes.

Anxiety and/or depression have been related to tobacco consumption and smoking cessation in distinct populations, including pregnant women [26,27,28,29,30]. Higher levels of anxiety and/or depression were found in pregnant women who smoke [28,30,31,32,33]. Likewise, a recent review found that psychological factors play an important role in smoking cessation, and not suffering from depression is one of the major predictors of quitting during pregnancy [34].

Despite the strong association between tobacco consumption and anxiety or depression during pregnancy, these factors have been concurrently considered a small number of times when their effects on maternal and neonatal health were studied. Few studies have focused on examining the joint effect of maternal tobacco consumption and anxiety or depression during pregnancy, and results are somewhat controversial. Regarding depression, although Schechter et al. [35] found that exposure to tobacco consumption has a higher negative effect on the neonate’s birth weight of mothers with co-occurring depression symptoms, no significant cumulative effect on the mother’s depression in the effect of smoking during pregnancy was found on premature birth or low birth weight by Quispel et al. [36]. Regarding anxiety, a strong relationship has been independently found between anxiety and smoking and premature birth [37,38]. However, to our knowledge, the only study that evaluated the simultaneous effect of maternal anxiety and tobacco consumption on pregnancy complications showed controversial results, and no significant effect was found between tobacco consumption and pregnancy complications [39].

In sum, tobacco consumption and anxiety or depression have been concurrently considered few times when effects were studied on maternal and neonatal health, despite their similar effects and strong associations.

It is also worth pointing out that most of the studies that evaluate the consequences of smoking during pregnancy on maternal and neonatal health focused on comparing non-smokers and smokers, leaving aside the possible effect of quitting smoking during pregnancy, and the few studies that evaluate this effect focus on the neonate’s weight [40,41]. To analyze the effects of quitting smoking on other aspects of maternal and neonatal health can be particularly relevant to promoting more effective health practices, specifically to the decision of health professionals to advise women to reduce or quit smoking during pregnancy, and to motivate pregnant women to quit smoking.

Taking this into consideration, this study aims to analyze the possible interaction effects between tobacco consumption during pregnancy (continuous, quitting and no consumption) and maternal anxiety or depression in the first trimester of pregnancy on maternal and neonatal health (pregnancy complications, delivery complications, neonatal health complications, gestational age and birth weight).

## 2. Materials and Methods

### 2.1. Procedure and Participants

The present research was conducted in accordance with the Helsinki Declaration and received previous approval from the Ethical Commission of all institutions involved (Ethical Board consent number 2010/299). This is a longitudinal study with three assessment waves: first trimester of pregnancy (M = 9.94 weeks; SD = 4.22), third trimester of pregnancy (M = 33.28 weeks; SD = 2.05), and 2 months postpartum (M = 7.39 weeks; SD = 0.86). Pregnant women attending primary public healthcare services in the northwest of Spain were recruited in the first trimester of pregnancy (*N* = 901). Women were eligible to participate if they were at least 18 years of age, were in the first trimester of pregnancy and spoke Spanish. The aims and procedures were explained and pregnant women who were willing to participate provided written informed consent. Two trained psychologists conducted the interviews individually. Women completed a questionnaire on socio-demographic variables, health status, and tobacco consumption, and self-reported measures of anxiety and depression. Maternal anxiety and depression and tobacco consumption status (continuous, quitting or no consumption) were considered in the first trimester of pregnancy, in accordance with recent evidence in the literature indicating that the major adverse effects on fetus health and development may occur in the first trimester of gestation [5]. The first trimester is also the moment in which pregnant women usually decide to quit smoking [28,42]. Tobacco consumption was assessed in the three assessment waves by self-reported and biochemical tests. In the first trimester, socio-demographic data were also collected, while health variables related to pregnancy were evaluated in the third trimester. Information regarding delivery and the neonate’s health was collected at 2 months postpartum.

**Figure 1:**
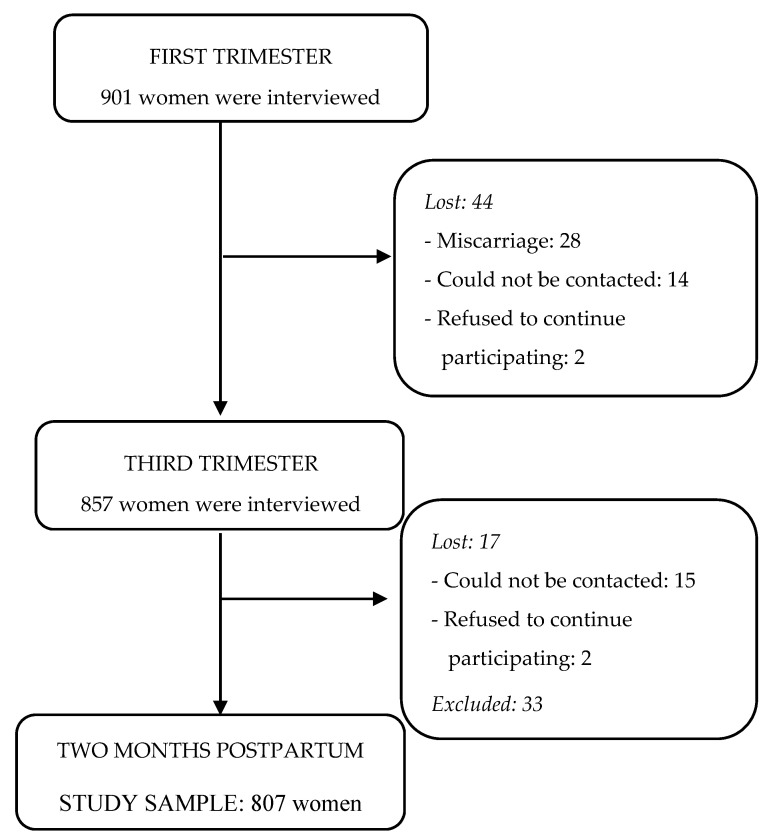
Flow diagram of recruitment and progress through study.

The sample comprises 807 pregnant women (see Figure 1). From the 901 pregnant women recruited in the first trimester of pregnancy, 94 women were lost (10.4%) at 2 months postpartum: 28 had suffered a miscarriage, 29 could not be contacted, 4 refused to continue participating in the study and 33 pregnant women were excluded (23 women for changing consumption status after first trimester of pregnancy and 10 for consuming other substances).

### 2.2. Measures

#### 2.2.1. Socio-Demographic, Health and Tobacco Consumption Information

A questionnaire was used to collect information regarding socio-demographic and pregnancy-related aspects, consumption information, pregnancy and delivery complications (e.g., threat of abortion, placental problems, threat of premature birth, hemorrhage, fetal suffering), neonate’s health problems (e.g., respiratory problems), gestational age at birth and birth weight.

#### 2.2.2. Consumption Status

Pregnant women were classified into three groups based on their self-report of tobacco consumption and the result of the biochemical test: continuous smokers, continuous quitters and non-smokers. Women who maintained their consumption throughout the pregnancy were classified as continuous smokers. Women who reported quitting when they expected or confirmed they were pregnant and who maintained abstinence during pregnancy were classified as continuous quitters. Women who reported no smoking in the past or who reported quitting at least one year before they became pregnant were classified as non-smokers. In the first evaluation, pregnant women were asked if they had ever been smokers and if they had smoked before pregnancy. If the answer was yes, they were requested to indicate the number of cigarettes per day they smoked before pregnancy; if the answer was negative, they were requested to report how long they had gone without smoking. At all times of the evaluation, they were asked what was their current situation in relation to tobacco consumption using multiple answer options: never smoker, currently I do not smoke and I have quit during pregnancy, I am an occasional smoker or I am a daily smoker. At the postpartum evaluation, an alternative answer was added: I quit smoking during pregnancy but started smoking again. Likewise, at all times, it was requested that those who indicated that they had quit smoking, specified in which month they quit, and those women who defined themselves as smokers, indicated the number of cigarettes they smoked. Because the aim of the study was the effects of tobacco consumption status (continuous, quitting or no consumption) during pregnancy on maternal and neonatal health, women reporting changes in their consumption status after the first trimester of pregnancy (*n* = 23) were not included in the analyses.

Self-reports of smoking abstinence were biochemically verified through urinary cotinine by Cotinine Test Medi-marketing (cut-off 200 ng/mL) in the first trimester and through carbon monoxide (CO) in expired air in the third trimester and at 2 months postpartum (cut-off of 5 ppm) using a Carbon Monoxide Monitor (model Smokerlyzer Pico Simple; Bedfont Technical Instruments Ltd., Kent, UK).

#### 2.2.3. Anxiety

The State Anxiety Scale (STAI-S) of the State–Trait Anxiety Inventory [43] was used to assess women’s anxiety in the first trimester of pregnancy. The STAI-S consists of a 20-item scale designed to assess the current state of anxiety. The score ranges from 0 to 60 and higher scores indicate higher anxiety symptoms. The STAI-S Spanish version showed good internal consistency and proposed a clinical cut-off >31 to screen for high-anxiety in women. In the present study, the STAI-S showed excellent internal consistency (α = 0.94).

#### 2.2.4. Depression

The Edinburgh Postnatal Depression Scale (EPDS) was used to assess women’s depression in the first trimester of pregnancy. The EPDS is a 10-item self-reported questionnaire designed to assess the intensity of depression symptoms within the previous 7 days [44]. The EPDS is the most widely used questionnaire to screen pregnancy and postpartum depression symptoms [28,45]. The EPDS Spanish version showed good internal consistency [46]. In the present study, the EPDS showed good internal consistency (α = 0.85).

### 2.3. Data Analysis

Chi-square tests were performed to analyze the associations and the interaction effects between tobacco consumption status, anxiety or depression on pregnancy, delivery and neonatal health complications (dichotomic dependent variables). The MANCOVA was performed to analyze the interaction effects between tobacco consumption status, anxiety or depression (independent variables) on gestational age and birth weight (continuous dependent variables). The sociodemographic variables associated with the independent variables (marital status, parity, educational level and previous consumption) were included in the models as covariates. Pairwise comparisons were applied to assess within-group differences.

Data were analyzed with IBM SPSS 23.0 Windows version (PASW Statistics for Windows, SPSS Inc., Chicago, IL, USA). Statistical significance level was considered at p < 0.05.

## 3. Results

### 3.1. Participant Sociodemographic Characteristics

Participants’ age ranged between 18 and 46 years old (M = 32.74 years; SD = 4.35) and most were married or cohabiting (83.8%). More than half had university studies (53.4%) and were employed (70.9%). In this sample, 28% smoked before pregnancy and their average of previous consumption was 10.88 (*SD* = 6.98). Most of the infants were the firstborn (65.9%), and were born with 37 weeks or more (96.0%) and with a normal weight (94.4%; M = 3274.99 g; SD = 547.50).

### 3.2. Associations between Tobacco Consumption, Anxiety or Depression, and Sociodemographic Characteristics

Significant associations were found between maternal tobacco consumption status and marital status, parity, educational level, professional status, and socioeconomic level. Significant associations were also found between maternal depression and educational level and between maternal anxiety and marital status (see Table 1).

### 3.3. Associations between Tobacco Consumption and Anxiety or Depression

Maternal tobacco consumption status was associated with anxiety (χ^2^ = 15.18, *p* = 0.001, Cramer’s V = 0.14) and depression (χ^2^ = 12.87, *p* = 0.002, Cramer’s V = 0.13) in the first trimester of pregnancy. Continuous smokers during pregnancy were more likely to present high anxiety (14.8% vs. 8.2% vs. 5.2%) and depression (18.8% vs. 9.2% vs. 8.3%) than continuous quitters and non-smokers. No significant differences were found between continuous quitters and non-smokers during pregnancy in the rates of high anxiety (Z = 0.3, *p* = 0.748) and depression (Z = 0.1, *p* = 0.929).

### 3.4. Associations between Tobacco Consumption, Anxiety or Depression, and the Mother’s and the Neonate’s Health

Maternal tobacco consumption status was associated with pregnancy (*p* = 0.014, Cramer’s V = 0.10) and delivery (*p* < 0.001, Cramer’s V = 0.21) complications and neonatal health complications (*p* = 0.025, Cramer’s V = 0.10). Continuous smokers during pregnancy were more likely to present pregnancy and delivery complications, and their neonates to present health complications than continuous quitters and non-smokers. No significant differences were found between continuous quitters and non-smokers on the rates of pregnancy (Z = 0.3, *p* = 0.781) and delivery (Z = 0.5, *p* = 0.583) complications, and neonatal health (Z = 0.5, *p* = 0.645) complications (see Table 2).

Significant differences were found in neonatal birth weight in accordance with maternal tobacco consumption status (*F*_(804)_ = 5.53, *p* = 0.004). Neonates of continuous smokers were born with lower weight than neonates of continuous quitters (*p* = 0.049) and neonates of non-smokers (*p* = 0.003), with no significant differences between the weight of neonates born to continuous quitters and non-smokers (3128.36 vs. 3294.39 vs. 3304.03). However, no significant differences were found in gestational age in accordance with tobacco consumption (*F*_(804)_ = 5.53, *p* = 0.315).

Regarding maternal anxiety and depression in the first trimester of pregnancy, high anxiety was associated with more delivery complications (*p* = 0.040, Cramer’s V = 0.07) and depression was associated with more pregnancy complications (*p* = 0.034, Cramer’s V = 0.08).

Significant differences were found in the neonate’s weeks of gestation at birth in accordance with the mother’s anxiety (*t*_(805)_ = 2.52, *p* = 0.012) and the mother’s depression in the first trimester (*t*_(805)_ = 1.35, *p* = 0.045). Neonates of high anxiety or depressed mothers were born with less weeks of gestation than neonates of low anxiety or non-depressed mothers. No differences were found in birth weight according to the mother’s anxiety (*t*_(805)_ = 0.37, *p* = 0.713) or depression (*t*_(805)_ = 0.70, *p* = 0.484) in the first trimester of pregnancy.

### 3.5. Interaction Effects between Tobacco Consumption Status and Anxiety or Depression on the Health of Mothers and Neonates

No significant interaction effects between maternal tobacco consumption status and anxiety were observed for pregnancy complications (χ^2^ = 9.31; *p* = 0.097).

Significant interaction effects between maternal tobacco consumption status and anxiety were observed for delivery complications (see Table 3). Continuous smokers with high anxiety (group 1) had more delivery complications than any other group (groups 2, 3, 4, 5 and 6). Continuous smokers with low anxiety (group 2) had more delivery complications than non-smokers with low anxiety (group 6). Both, continuous smokers with low anxiety (group 2) and continuous quitters with high or low anxiety (groups 3 and 4), had more delivery complications than non-smokers with high anxiety (group 5).

Significant interaction effects between maternal tobacco consumption status and anxiety were also observed for neonatal health complications. Neonates of continuous smokers with high anxiety (group 1) had more health complications than neonates of non-smokers, regardless of their level of anxiety (groups 5 and 6).

Significant interaction effects between maternal tobacco consumption status and anxiety were observed for gestational age at birth (see Table 4). Neonates of continuous smokers with high anxiety (group 1) were born after less gestational weeks than neonates of non-smokers with low anxiety (group 6). No significant interaction effect between maternal tobacco consumption status and anxiety were observed for birth weight (*F*_(802)_ = 0.30, *p* = 0.596).

Significant interaction effects between maternal tobacco consumption status and depression were observed for pregnancy complications (see Table 3). Non-depressed continuous smokers (group 2) had more pregnancy complications than non-depressed continuous quitters (group 4) and non-depressed non-smokers (group 6).

Significant interaction effects between maternal tobacco consumption status and depression were observed for delivery complications. Depressed continuous smokers (group 1) had more delivery complications than depressed (group 5) and non-depressed (group 6) non-smokers. Non-depressed continuous quitters (group 4) had more delivery complications than depressed non-smokers (group 5). No significant interaction effects between maternal tobacco consumption status and depression were observed for neonatal health complications (χ^2^ = 9.63; *p* = 0.087).

Likewise, significant interaction effects between maternal tobacco consumption status and depression were observed for weeks of gestation at birth (see Table 4). Neonates of depressed continuous smokers (group 1) were born after less gestational weeks than neonates of non-depressed non-smokers (group 6). No significant interaction effects between maternal tobacco consumption status and depression were found on birth weight (*F*_(802)_ = 1.08, *p* = 0.398).

## 4. Discussion

This study aimed to analyze the possible interaction effects between tobacco consumption during pregnancy and maternal anxiety or depression in the first trimester of pregnancy on the health of mothers and newborns. An association between maternal tobacco consumption and anxiety or depression in the first trimester of pregnancy was found. Maternal tobacco consumption status was associated with high anxiety as well as with depression, as previously noted in the literature [28,30,31,32,33]. Continuous smokers were more likely to present high anxiety and depression than continuous quitters and non-smokers in the first trimester of pregnancy. On the other hand, no significant differences were found between continuous quitters and non-smokers on rates of anxiety or depression. These results highlighted the possible beneficial impact of quitting smoking during pregnancy for the prenatal mental health of mothers.

An independent effect of maternal tobacco consumption, anxiety and depression during pregnancy on the health of mothers and neonates was found. Maternal tobacco consumption during pregnancy was associated with more pregnancy and delivery and neonatal complications, including low birth weight. Interestingly, maternal anxiety in the first trimester of pregnancy was associated with delivery complications, while maternal depression in the first trimester of pregnancy was associated with pregnancy complications.

Several studies have shown that tobacco consumption during pregnancy directly affects the health of both mothers and neonates throughout the perinatal period [2,3,4,5,6,7,8,9,47,48]. Tobacco consumption during pregnancy can lead to epigenetic changes that affect the health of mothers and neonates. One of the main mechanisms is through the impact of tobacco carbon monoxide. This component of tobacco can cause defective vascularization, placental hypertrophy and/or local hypoxia, which reduces uterine blood flow, increasing the risk of complications and restricting fetal growth [3]. Another important action mechanism is the effect of nicotine on the organism. This component has a vasoconstrictor effect and has also been related to the activation of the enzyme phospholipase A2, which is associated with undergoing abortions. However, during the postpartum period, the effects of tobacco on health do not exclusively affect the mother, as the nicotine can be transferred to the neonate through breast milk [11]. These data emphasize the negative effect of tobacco consumption on the course of pregnancy. Contrarily, no significant differences were found between continuous quitters and non-smokers on the studied maternal health complications, neither on neonatal weight at birth nor health complications. These results suggest that early and continued tobacco quitting during pregnancy could decrease maternal risks of suffering health problems during this period of life [40,41]. It should be noted that results (56.6% of women continue to smoke during pregnancy) indicate higher prevalence of smoking than other research carried out in Spain previously (27–42%) [49,50], although similar to the data provided by studies carried out in Europe (57–65%) [51,52]. These differences between prevalence may be due to the methodologies used. Spanish studies are retrospective and based on self-reports (methodology susceptible to bias due to social desirability), whereas the European ones are prospective and use biochemical validation of consumption. As with tobacco consumption, several studies have shown that maternal anxiety and/or depression negatively affect the health of both mothers and neonate throughout the perinatal period [15,16,17,18]. This study added that maternal anxiety and depression are specifically associated with different health complications and at different times. Maternal anxiety was found to be associated with complications related to delivery, while maternal depression seems to affect health complications arising during pregnancy. These results contribute to clarify the potential differential impact of maternal anxiety and depression on pregnancy and delivery complications, an issue that remained unclear in the literature [19]. Different underlying epigenetic and endophenotypic mechanisms were suggested to explain the effect of maternal anxiety and/or depression on the emergence of different types of complications. Several studies pointed out that the presence of anxiety or depression can be a stressor for pregnant women, stimulating the activation of different mechanisms, namely the hypothalamic axis (HPA), affecting the course of pregnancy [15,53]. Likewise, it could also affect the physiological development of the newborn by interfering with the levels of cortisol, norepinephrine and dopamine [16]. Experiencing high levels of anxiety or depression during pregnancy may contribute to an increase in hormones such as cortisol and catecholamines, which cause changes in immunologic functioning and uterine blood flow during human pregnancy, thus increasing vulnerability [18].

The results of the interaction effect between maternal consumption status and anxiety or depression suggested that different kinds of interactions were also found. Specifically, three different kinds of interactions were found. First, an interaction in which the fundamental role is of tobacco consumption, such as the results of the interaction effect between maternal consumption status and anxiety or depression on delivery complications. Second, an interaction in which the fundamental role is developed by anxiety or depression. Regarding neonatal health problems, the results of the interaction effect between maternal consumption status and anxiety or depression suggested that effects of tobacco consumption on the gestational age of neonates at birth only emerged when maternal consumption status was associated with anxiety or depression, as no independent effects of maternal consumption status were found. The neonates of continuous smokers with high anxiety or depression were born with fewer weeks than the neonates of non-smokers with no anxiety or no depression.

Finally, an interaction that suggested a cumulative effect of maternal anxiety in the first trimester of pregnancy on the effects of tobacco consumption status (continuous, quitting and no consumption) during pregnancy on the mother’s health was also found. Continuous smokers with high-anxiety presented more delivery complications than continuous smokers with low-anxiety. This effect could be explained by the simultaneous activation of the mechanisms activated by tobacco consumption [3,11] and those activated due to the presence of anxiety [15,16,17,18]. However, no cumulative effects of anxiety or depression were found on pregnancy complications. This result suggested that the effect of maternal tobacco consumption can be so adverse that anxiety and depression did not increase the risks of pregnancy complications.

Altogether, these data highlighted the close relationship between tobacco consumption, the presence of psychological symptoms (anxiety or depression) and adverse health outcomes [39], being one of the few studies that jointly assess the effect of maternal tobacco consumption, anxiety or depression on health during pregnancy.

This study has certain limitations. Although the selected measures are those of international reference and have shown good internal consistency, anxiety and depression were evaluated with self-reports, which may imply different biases of social desirability, such as the concealment of symptoms. Likewise, pregnancy and delivery complications were evaluated dichotomously (presence or absence of complications), what can lead to bias in the interpretation of results and which does not allow for an in-depth study of these repercussions. In future studies, it would be relevant not only to assess the presence of complications, but also the number, type and severity of the same. Likewise, the sample was collected exclusively in public healthcare centers, which does not allow for the generalization of the results to all pregnant women, since women who attend private healthcare facilities could present different characteristics. Despite these limitations, this study presents several strengths. This is a longitudinal study that followed a large number of women during pregnancy and the early postpartum period. This allowed exploring possible interaction effects between the variables. Moreover, the cumulative effect of anxiety or depression in the effect of tobacco consumption was studied in different relevant aspects related to maternal and infant health in pregnancy and the early postpartum. Moreover, self-reports of tobacco abstinence were validated with biochemical tests. This is particularly relevant in this population due to the social pressure suffered by women not to smoke [54] and this may suppose that some women hide their consumption [55].

The results of this research have practical implications and could help to improve healthcare during pregnancy, as they highlight the importance of evaluating both the presence of tobacco consumption and the level of anxious and depressive symptoms during pregnancy. One of the main reasons for health professionals not to advise pregnant women to quit tobacco is the consideration that anxiety can increase after quitting smoking. However, quitting smoking reduces the risk of suffering anxiety and depression during pregnancy, as well as health risks. These data are proof of how quitting smoking can be beneficial both for the physical and psychological health of pregnant women as well as for the newborn itself. Data highlights the importance of advice during the perinatal period focusing on smoking cessation and not reducing cigarette consumption, since the former would lead to a lower prevalence of health problems (physical and psychological) in both women as well as their children.

## 5. Conclusions

This study suggests that there are different kinds of interaction effects between tobacco consumption and anxiety or depression on the health of mothers. The likelihood of health problems increases when mothers jointly present tobacco consumption and high anxiety. Specifically, a cumulative effect of anxiety and tobacco consumption on delivery complications was found. Likewise, a beneficial effect of quitting smoking was found both on the physical health of newborns and on the physical and psychological health of women. In particular, quitting smoking during pregnancy reduced the risk of suffering anxiety, depression and health complications.

## Figures and Tables

**Table 1:** Maternal socio-demographic characteristics, consumption status, anxiety and depression.

Socio-Demographic Characteristics	Mothers (*N* = 807)	Consumption Status				Anxiety			Depression		
	*%*	Continuous Smoker *n* = 128	Continuous Quitter *n* = 98	Non- Smoker *n* = 581	χ^2^	Anxiety *n* = 57	No Anxiety *n* = 750	χ^2^	Depression *n* = 81	No Depression *n* = 726	χ^2^
**Age**					1.56			0.10			0.64
18–34	63.3	64.8	68.4	62.1		61.4	63.5		59.3	63.8	
≥35	36.7	35.2	31.6	37.9		38.6	36.5		40.7	36.2	
**Marital status**					7.71 *			8.33 *			22.26 ***
Single/divorced	16.2	24.2	17.3	14.3		29.8	15.2		34.6	14.2	
Married/living with partner	83.8	75.8	82.7	85.7		70.2	84.8		65.4	85.8	
**Parity**					10.35 *			0.56			3.34
Primiparous	65.9	68.0	79.6	63.2		61.4	66.3		56.8	66.9	
Multiparous	34.1	32.0	20.4	36.8		38.6	33.7		43.2	33.1	
**Educational level**					55.74 ***			1.50			6.99 *
Non-university (≤12 years)	46.6	75.8	50.0	39.6		54.4	46.0		60.5	45.0	
University (>12 years)	53.4	24.2	50.0	60.4		45.6	54.0		39.5	55.0	
**Professional status**					7.31 *						0.39
Unemployed	29.1	39.1	26.5	27.4		31.6	28.9	0.18	32.1	28.8	
Employed	70.9	60.9	73.5	72.6		68.4	71.1		67.9	71.2	
**Socioeconomic level**					9.35 *			0.98			0.01
Medium-low	62.8	73.5	70.1	59.6		69.2	62.3		64.4	62.7	
Medium-high	37.2	26.5	29.9	40.4		30.8	37.7		35.6	37.3	

* *p* < 0.05; *** *p* < 0.001.

**Table 2:** Association between consumption status, anxiety or depression, and health complications.

Health Complications	Consumption Status					Anxiety			Depression			
	Continuous Smoker (1)	Continuous Quitter (2)	Non-Smoker (3)	χ^2^	Group Comparisons	Anxiety	No Anxiety	χ^2^	Depression	No Depression	χ^2^	Total
Pregnancy complications	21.1	9.2	12.4	8.55 *	1 > 2,3	15.8	13.2	0.31	21.0	12.5	4.49 *	13.4
Delivery complications	27.3	13.3	8.3	36.64 ***	1 > 2,3	19.3	11.3	3.2 *	9.9	12.1	0.35	11.9
Neonatal health complications	12.5	10.2	6.0	7.41 *	1 > 3	7.0	7.6	0.03	6.2	7.7	0.25	7.6

* p < 0.05; *** p < 0.001.

**Table 3:** Interaction effects between consumption status and anxiety or depression on health complications.

Health Complications	Continuous Smoker		Continuous Quitter		Non-Smoker			
	Anxiety *n* = 19 (1)	No Anxiety *n* = 109 (2)	Anxiety *n* = 8 (3)	No Anxiety *n* = 90 (4)	Anxiety *n* = 30 (5)	No Anxiety *n* = 551 (6)	χ^2^	Group Comparison
Pregnancy complications	26.3	20.2	25.0	8.9	10.0	12.5	9.31	
Delivery complications	52.6	22.0	12.5	13.3	3.3	8.7	49.71 ***	1 > 2,3,4,5,6 2 > 6 2,3,4 > 5
Neonatal health complications	21.1	11.0	12.5	11.1	3.2	6.4	12.69 *	1 > 5,6
**Health Complications**	**Depression** ***n* = 24** **(1)**	**No Depression** ***n* = 104** **(2)**	**Depression** ***n* = 9** **(3)**	**No Depression** ***n* = 89** **(4)**	**Depression** ***n* = 48** **(5)**	**No Depression** ***n* = 533** **(6)**	**χ^2^**	**Group Comparison**
Pregnancy complications	25.0	20.2	22.2	9.0	20.8	11.6	12.18 *	2 > 4,6
Delivery complications	25.0	26.9	11.1	13.5	2.1	9.0	35.22 ***	1 > 5,6 4 > 5
Neonatal health complications	8.3	13.5	11.1	11.2	8.3	6.0	9.63	

* *p* < 0.05; *** *p* < 0.001.

**Table 4:** Interaction of consumption status and anxiety or depression on weeks and weight at birth.

	Continuous Smoker		Continuous Quitter		Non-Smoker			
Weeks and Weight at Birth	Anxiety (1) M (SD)	No Anxiety (2) M (SD)	Anxiety (3) M (SD)	No Anxiety (4) M (SD)	Anxiety (5) M (SD)	No Anxiety (6) M (SD)	F	Group Comparison
Weeks of gestation	38.48	39.34	38.25	39.37	39.27	39.43	6.90 *	1 < 6
	(3.37)	(1.34)	(1.91)	(1.41)	(1.23)	(1.50)		
Birth weight	3066.84	3139.08	3273.75	3296.22	3358.33	3301.39	0.30	
	(546.93)	(455.64)	(526.01)	(550.85)	(623.25)	(556.25)		
**Weeks and Weight at Birth**	**Depression** **(1)**	**No** **Depression** **(2)**	**Depression** **(3)**	**No** **Depression** **(4)**	**Depression** **(5)**	**No Depression (6)**	**F**	**Group Comparison**
Weeks of gestation	38.40	39.11	38.70	39.38	39.21	39.44	6.21 *	1 < 6
	(1.17)	(1.95)	(1.95)	(1.40)	(1.32)	(1.50)		
Birth weight	3075.77	3138.68	3321.00	3287.28	3299.58	3304.75	1.08	
	(485.40)	(472.15)	(490.04)	(545.49)	(575.96)	(556.40)		

* *p* < 0.05.

## References

[1] Homer L., Bernard C., Collet M. (2014). Management and outcomes of pregnancies complicated by preterm premature rupture of membranes before 26 weeks of gestation. Gynécol. Obstet. Fertil..

[2] Héquet D., Ricbourg A., Sebbag D., Rossignol M., Lubrano S., Barranger E. (2013). Placenta accreta: Screening, management and complications. Gynécol. Obstet. Fertil..

[3] Phelan S. (2014). Smoking cessation in pregnancy. Obstet. Gynecol. Clin. N. Am..

[4] Shobeiri F., Jenabi E. (2017). Smoking and placenta previa: A meta-analysis. J. Matern. Fetal Neonatal Med..

[5] Escobar-Padilla B., Perez-López C.A., Martínez-Puon H. (2017). Risk factors and clinical features of ectopic pregnancy. Rev. Med. Inst. Mex. Seguro Soc..

[6] Hyland A., Piazza K., Hovey K., Ockene J., Andrews C., Rivard C., Wactawski-Wende J. (2015). Associations of lifetime active and passive smoking with spontaneous abortion, stillbirth and tubal ectopic pregnancy: A cross-sectional analysis of historical data from the women´s health initiative. Tob. Control.

[7] U.S. Department of Health and Human Services (2014). The Health Consequences of Smoking—50 Years of Progress: A Report of the Surgeon General.

[8] Lee S.W., Han Y.J., Cho D.H., Kwak H.S., Ko K., Park M.H., Han J.Y. (2018). Smoking exposure in early pregnancy and adverse pregnancy outcomes: Usefulness of Urinary Tobacco-Specific Nitrosamine Metabolite 4-(Methylnitrosamino)-1-(3-Pyridyl)-1-Butanol Levels. Gynecol. Obstet. Investig..

[9] Levy D., Jiang M., Szklo A., Almeida L.M., Autran M., Bloch M. (2013). Smoking and adverse maternal and child health outcomes in Brazil. Nicotine Tob. Res..

[10] Ion R., Bernal A.L. (2015). Smoking and preterm birth. Reprod. Sci..

[11] U.S. Department of Health and Human Services (2001). Women and Smoking: A Report of the Surgeon General.

[12] Timur S., Hotun N., Omac M. (2017). Maternal smoking and newborn sex, birth weight and breastfeeding: A population-based study. J. Matern. Fetal Neonatal Med..

[13] Bruin J.E., Gerstein H.C., Holloway A.C. (2010). Long-term consequences of fetal and neonatal nicotine exposure: A critical review. Toxicol. Sci..

[14] Jaddoe V., van Duijn C., van der Heiden A., Makenbach J., Moll H., Steegers E., Hofman A. (2010). The Generation R Study: Design and cohort update 2010. Eur. J. Epidemiol..

[15] Bayrampour H., McDonald S., Tough S. (2015). Risk factors of transient and persistent anxiety during pregnancy. Midwifery.

[16] Diego M., Field T., Hernandez-Reif M., Cullen C., Schanberg S., Kuhn C. (2009). Prepartum, postpartum, and chronic depression effects on newborns. Psychiatry.

[17] Ding X.X., Wu Y.L., Xu S.J., Zhu R.P., Jia X.M., Zhang S.F., Huang K., Zhu P., Hao J.H., Tao F.B. (2014). Maternal anxiety during pregnancy and adverse birth outcomes: A systematic review and meta-analysis of prospective cohort studies. J. Affect. Disord..

[18] Kelly R., Russo J., Holt V., Danielsen B., Zatzick D., Walker E., Katon W. (2002). Psychiatric and substance use disorders as risk factors for low birth weight and preterm delivery. Obstet. Gynecol.

[19] Alder J., Fink N., Bitzer J., Hösli L., Holzgreve W. (2007). Depression and anxiety during pregnancy: A risk factor for obstetric, fetal and neonatal outcome? A critical review of the literature. J. Matern. Fetal Neonatal Med..

[20] Grote N., Bridge J., Gavin A., Melville J., Iyengar S., Katon W. (2010). A meta-analysis of depression during pregnancy and the risk of preterm birth, low birth weight and intrauterine growth restriction. Arch. Gen. Psychiatry.

[21] Staneva A., Bogossian F., Pritchard M., Wittkoeski A. (2015). The effects of maternal depression, anxiety and perceived stress during pregnancy on preterm birth: A systematic review. Women Birth.

[22] Liu Y., Murphy S.K., Murtha A.P., Fuemmeler B.F., Schildkraut J., Huang Z., Overcash F., Kurtzberg J., Jirtle R., Iversen E.S. (2012). Depression in pregnancy, infant birth weight and DNA methylation of imprint regulatory elements. Epigenetics.

[23] Fransson E., Ortenstrand A., Hjelmstedt A. (2011). Antenatal depressive symptoms and preterm birth: A prospective study of a Swedish national sample. Birth.

[24] Doktorchik C., Premji S., Slater D., Wiliamson T., Touh S., Patten S. (2018). Patterns of change in anxiety and depression during pregnancy predict preterm birth. J. Affect. Disord..

[25] Shapiro G.D., Séquin J.R., Muckle G., Monnier P., Fraser W.D. (2017). Previous pregnancy outcomes and subsequent pregnancy anxiety in a Quebec Prospective cohort. J. Psychosom. Obstet. Gynecol..

[26] Hauge L.J., Torgersen L., Vollrath M. (2012). Associations between maternal stress and smoking: Findings from a population-based prospective cohort study. Addiction.

[27] Irfan M., Hague A.S., Shahzad H., Samani Z.A., Awan S., Khan J.A. (2016). Reasons for failure to quit: A cross-sectional survey of tobacco use in major cities in Pakistan. Int. J. Tuberc. Lung Dis..

[28] Míguez M.C., Pereira B., Figueiredo B. (2017). Tobacco consumption and spontaneous quitting at the first trimester of pregnancy. Addict. Behav..

[29] Mykletun A., Overland S., Edvard L., Liabo H.M., Stewart R. (2008). Smoking in relation to anxiety and depression: Evidence from a large population survey: The HUNT study. Eur. Psychiatry.

[30] Tong L., Shi H.L., Zhang Z., Yuan Y., Xian Z.J., Jiang X.X., Xiong X. (2016). Mediating effect of anxiety and depression on the relationship between Attention-deficit/hyperactivity disorder symptoms and smoking/drinking. Sci. Rep..

[31] Maxson P., Edwars S., Ingram A., Miranda M.L. (2012). Psychosocial differences between smokers and non-smokers during pregnancy. Addict. Behav..

[32] Orr S.T., Blazer D.G., Orr C.A. (2012). Maternal prenatal depressive symptoms, nicotine addiction, and smoking-related knowledge, attitudes, beliefs, and behaviors. Matern. Child Health J..

[33] Smedberg J., Lupattelli A., Mardby A.C., Overland S., Nordeng H. (2015). The relationship between maternal depression and smoking cessation during pregnancy—A cross-sectional study of pregnant women from 15 European countries. Arch. Womens Ment. Health.

[34] Riaz M., Lewis S., Naughton F., Ussher M. (2018). Predictors of smoking cessation during pregnancy: A systematic review and meta-analysis. Addiction.

[35] Schechter J., Do E., Zhang J., Hoyo C., Murphy S., Kollins S., Fuemmeler B. (2018). Effect of prenatal smoke exposure on birth weight: The moderating role of maternal depressive symptoms. Nicotine Tob. Res..

[36] Quispel C., Bangma M., Kazemier B.M., Steeger E.A., Hoogendijk W.J., Papatsonis D., Paarlberg K.M., Lambregtse-Van Den Berg M.P., Bonsel G.J. (2014). The role of depressive symptoms in the pathway of demographic and psychosocial risks to preterm birth and small for gestational age. Midwifery.

[37] Grimstad H., Schei B., Backe B., Jacobsen G. (1999). Anxiety, psysical abuse and low birth weight. Scand. J. Public Health.

[38] Pavlov M., Steiner N., Kessous R., Weintraun A.Y., Sheiner E. (2014). Obstetric and neonate outcome in patients with anxiety disorders. J. Matern. Fetal Neonatal Med..

[39] Da Costa D., Brender W., Larouche J. (1998). A prospective study of the impact of psychosocial and lifestile variables on pregnancy complications. J. Psychosom. Obstet Gynaecol..

[40] Benjamin-Garner R., Stotts A. (2013). Impact of smoking exposure change on infant birth weight among a cohort of women in a prenatal smoking cessation study. Nicotine Tob. Res..

[41] Rode L., Kiaergaard H., Damm P., Ottensen B., Hegaard H. (2013). Effect of smoking cessation on postpartum weight gain and neonatal birth weight. Obstet. Gynecol..

[42] Schneider S., Huy C., Schütz J., Diehl K. (2010). Smoking cessation during pregnancy: A systematic literature review. Drug Alcohol Rev..

[43] Spielberger C.D., Gorsuch R.L., Lushene R. (1970). Manual for the State-Trait Anxiety Inventory.

[44] Cox J.L., Holden J.M., Sagovsky R. (1987). Detection of postnatal depression: Development of the 10-item Edinburgh Postnatal Depression Scale. Br. J. Psychiatry.

[45] Figueiredo B., Conde A. (2011). Anxiety and depression in women and men from early pregnancy to 3-months postpartum. Arch. Womens Ment. Health.

[46] Vázquez M.B., Míguez M.C. (2019). Validation of the Edinburgh postnatal depression scale as a screening tool for depression in Spanish pregnant women. J. Affect. Disord..

[47] Chelchiwska M., Gajewska J., Maciejewski T.M., Mazur J., Oltarzewski M., Ambroszkiewicz J. (2020). Associations between Maternal and Fetal Levels of Total Adiponectin, High Molecular Weight Adiponectin, Selected Somatomedins, and Birth Weight of Infants of Smoking and Non-Smoking Mothers. Int. J. Environ. Res. Public Health.

[48] Míguez M.C., Pereira B. (2020). Repercusiones del consumo de tabaco activo y/o pasivo en el embarazo y postparto. [Effects of active and/or passive smoking during pregnancy and the postpartum period]. Anales Pediatr..

[49] Cano J., Sánchez M., García M.C., Rabadán B., Yep G. (2007). Tabaquismo durante el embarazo en mujeres de la Comunidad de Madrid. [Smoking during pregnancy in women of Madrid]. Prev. Table.

[50] Martínez-Frías M.L., Rodríguez E., Bermejo E., Grupo Periférico del ECEMC (2005). Consumo de tabaco durante el embarazo en España: Análisis por años, comunidades autónomas y características maternas. [Tobacco smoking during pregnancy in Spain: An analysis according to years, autonomous communities and mother characteristics]. Med. Clin..

[51] Orton S., Bowker K., Cooper S., Naughton F., Ussher M., Pickett K., Leonardi-Bee J., Sutton S., Dhalwani N., Coleman T. (2014). Longitudinal cohort survey of women’s smoking behaviour and attitudes in pregnancy: Study methods and baseline data. BMJ.

[52] Balwicki L., Zarzecna-Baran M., Wierucki L., Jedrzejczyk T., Strahl M., Wrotkowska M., Goniewicz M.L., Zdrojewski T. (2016). Smoking among pregnant women in small towns in Poland. Int. J. Public Health.

[53] Pinto T.M., Caldas F., Nogueira-Silca C., Figueiredo B. (2017). Maternal depression and anxiety and fetal-neonatal growth. J. Pediatr..

[54] Wigginton B., Lee C. (2013). Stigma and hostility towards pregnant smokers: Does individuating information reduce the effect?. Psychol. Health.

[55] Míguez M.C., Pereira B. (2018). Prevalence of smoking in pregnancy: Optimization of the diagnosis. Med. Clín..

